# Tetralogy of Fallot Associated With Invasive Adrenocortical Tumor in an Adult Woman

**DOI:** 10.5812/ijem.3769

**Published:** 2012-04-20

**Authors:** Efren Martinez-Quintana, Fayna Rodriguez-Gonzalez, Maria Pino Alberiche-Ruano, Maria Soledad Martinez-Martin

**Affiliations:** 1 Cardiology Service. Insular-Materno Infantil University Hospital, Las Palmas de Gran Canaria, Spain; 2 Ophthalmology Service. Dr. Negrín University Hospital, Las Palmas de Gran Canaria, Spain; 3 Endocrinology Service. Insular-Materno Infantil University Hospital, Las Palmas de Gran Canaria, Spain; 4 Anatomopathology Service. Insular-Materno Infantil University Hospital, Las Palmas de Gran Canaria, Spain

**Keywords:** Tetralogy of Fallot, Adernal Cortex Neoplasms, Woman

## Abstract

Migration of cardiac neural crest cells into the pharyngeal arches and the pharyngeal and splanchnic mesoderm contributes to the development of the cardiac outflow tract. The adrenal cortex is derived from the splanchnic mesoderm. Neuroblastoma is more prevalent in patients with congenital heart disease than in the general population, because both originate from embryonal neural crest-derived cells. Similarly, and in light of recent embryological findings, abnormal development or migration of splanchnic mesoderm, possibly due to an underlying genetic defect, could contribute to the association of adrenocortical carcinoma and tetralogy of Fallot. We present the case of a cardiologically asymptomatic 49-year-old woman with total correction of tetralogy of Fallot in the first year of life.

## 1. Introduction

The conditions considered in the differential diagnosis of retroperitoneal masses include benign tumors (e.g., lipoma, teratoma, paraganglioma, or nerve sheath tumors such as schwannomas and neurofibromas), malignant neoplasms (e.g., sarcomas such as liposarcoma, leiomyosarcoma, and malignant fibrous histiocytoma; extragonadal germ cell tumors; lymphoma; renal cell carcinomas; or testicular tumors), pyogenic abscesses, hematomas, or the Budd–Chiari syndrome. Most primary retroperitoneal tumors arise from one or more of the mesenchymal tissues of the retroperitoneum, and others are derived from neuroectodermal elements or from remnants of the urogenital ridge. At present, adrenocortical carcinoma, a rare and heterogeneous malignancy, has a poor prognosis, and its pathology is incompletely understood (1).

## 2. Case Report

We present the case of a cardiologically asymptomatic 49-year-old woman with total correction of tetralogy of Fallot in the first year of life, to relieve the right ventricular outflow tract stenosis and repair the ventricular septal defect with a Gore-Tex patch. The patient had no cardiovascular risk factors and was not undergoing any cardiac treatment. She was treated with progesterone during the last 3 years because of menstrual disorders. During the last few months, she complained of pain in her right flank, which was associated with weight loss. The patient had no history of hypertension, headaches, palpitations, facial flushing, hirsutism, or Cushing’s syndrome.

**Figure 1:**
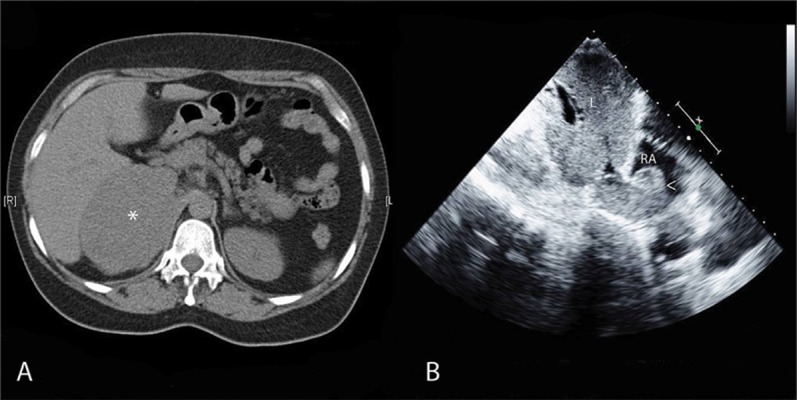
Computed Tomography Scan and Echocardiography Showing an Invasive Adrenocortical Carcinoma. A: Cross-sectional computed tomography scan showing a heterogeneous, solid mass measuring 9.3 × 7.4 cm in the right adrenal gland (asterisk), which was in close contact with the liver, the right kidney, and the inferior vena cava. B: Subcostal echocardiographic view showing infiltration of the inferior vena cava and a presumably metastatic mass in the right atrium (arrowhead). L: liver, RA: right atrium.

**Figure 2:**
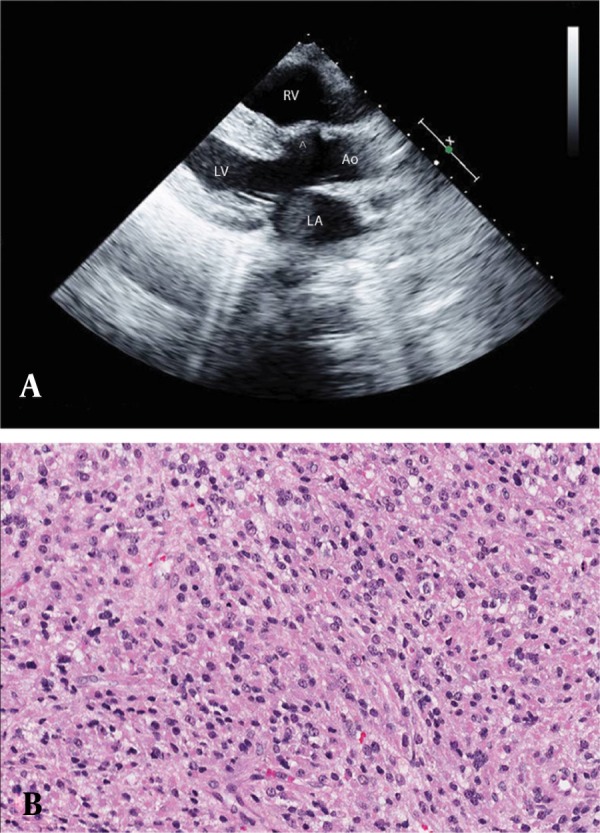
Echocardiogram of the Corrected Tetralogy of Fallot and Biopsy of the Adrenocortical Carcinoma. A: Long-axis echocardiographic view with an overriding aorta between both ventricles and an interventricular septal defect corrected with a patch (arrowhead). B: Hematoxylin and eosin stain showing an adrenocortical carcinoma with hyperchromasia, diffuse nuclear pattern of growth, and low mitotic activity. LA: left atrium, LV: left ventricle, RV: right ventricle; Ao: aorta. A color image is available in the online version of the article.

Abdominal ultrasonography and computed tomography (Figure 1A) showed the presence of a heterogeneous solid mass measuring 9.3 × 7.4 cm in the right adrenal gland; this mass was in contact with the liver, the right kidney, and the inferior vena cava. Echocardiography showed infiltration of the inferior vena cava with a tumor mass in the right atrium (Figure 1B) and an overriding aorta with no residual shunt in the repaired ventricular septal defect (Figure 2A). Laboratory test results showed increased levels of free testosterone (8.4 pcg/ml; normal range, [0.7–3.6]) and 17-hydroxyprogesterone (3.5 ng/ml; normal range, [0.15–1.1]). The levels of urine and plasma metanephrines, urine free cortisol, dehydroepiandrosterone (DHEA), basal cortisol, adrenocorticotropic hormone (ACTH), aldosterone, and plasma renin activity were within the normal ranges.

Exploratory laparotomy and biopsies of the adrenal tumor revealed an adrenocortical carcinoma (Figure 2B), which showed positive immunohistochemical staining for vimentin, synaptophysin, and inhibin, and negative staining for cytokeratin, epithelial membranous antigen (EMA), and CD10. The adrenal glands are composed of 2 major components: the adrenal medulla and the adrenal cortex. The adrenal cortex is derived from the splanchnic mesoderm, and the adrenal medulla is derived from the ectodermal chromaffin cells from the neural crest (2). The adrenocortical carcinoma, which is derived from the adrenal cortex, is an aggressive cancer, with an annual incidence of 1–2 per million population and a bimodal distribution, with peaks of incidence at about 5 years of age and again at 40 to 50 years of age. Patients can present either with a hormonal syndrome or with general symptoms caused by an abdominal mass (3). Although the pathogenesis of adrenocortical carcinoma is unknown, smoking has been reported to be a risk factor in men, and use of oral contraceptives in women, especially before the age of 25 years. However, no associations were observed with alcohol use, height, weight, or diet patterns for either sex (4).

An overriding aorta is a congenital malalignment defect, wherein the aorta fails to wedge between the mitral and tricuspid atrioventricular valves. In this situation, the aorta, which should receive all the blood from the left ventricle, overrides the ventricular septum to a greater (double-outlet right ventricle) or lesser (tetralogy of Fallot) extent, thereby receiving a proportionate amount of blood from the right ventricle.

There are several case reports of patients with coexisting neuroblastoma and congenital heart defects (2, 5-7), suggesting that abnormal migration and development of neural crest cells may be a common link between cardiac malformations and congenital neuroblastomas. Some studies suggest a higher prevalence of congenital heart defects among patients with neuroblastoma than among the general population (8-10); however, others have shown no such association (11). Additionally, autopsy findings in a 21-year-old male with a complete transposition of the great arteries revealed that congenital heart defects (11) are associated with adrenocortical carcinomas (12). The association between neuroblastoma, an embryonal cancer of the postganglionic sympathetic nervous system that mostly arises from the adrenal medulla, and congenital heart disease is considered plausible because a neuroblastoma originates from embryonal neural crest-derived cells, and these neural crest-derived cells are also essential in cardiogenesis (9, 13). However, recent embryological findings show that the outflow ventricular tract, which is involved in tetralogy of Fallot, is elongated by not only cardiac neural crest cells but also cardiogenic cells from the pharyngeal and splanchnic mesoderm (14); therefore, it seems plausible that adrenocortical carcinoma is associated with overriding malalignment defects (probably due to an underlying genetic defect). However, further investigations should be conducted to understand the correlation between adrenocortical tumors and congenital heart disease, in order to rule out other clinical and genetic risk factors that could play an important role in these findings.
